# A desire for distraction: uncovering the rates of media multitasking during online research studies

**DOI:** 10.1038/s41598-023-27606-3

**Published:** 2023-01-16

**Authors:** Allison C. Drody, Effie J. Pereira, Daniel Smilek

**Affiliations:** Department of Psychology, University of Waterloo, Waterloo, ON Canada

**Keywords:** Psychology, Human behaviour

## Abstract

Interpretations of task performance in many cognitive studies rest on the assumption that participants are fully attentive to the tasks they agree to complete. However, with research studies being increasingly conducted online where monitoring participant engagement is difficult, this assumption may be inaccurate. If participants were found to be engaging in off-task behaviours while participating in these studies, the interpretation of study results might be called into question. To investigate this issue, we conducted a secondary data analysis across nearly 3000 participants in various online studies to examine the prevalence of one form of off-task behaviour: media multitasking. Rates of media multitasking were found to be high, averaging 38% and ranging from 9 to 85% across studies. Our findings broadly raise questions about the interpretability of results from online studies and urge researchers to consider the likelihood that participants are simultaneously engaging in off-task behaviours while completing online research tasks.

In recent years, particularly during the pandemic, studies examining aspects of human cognition have increasingly been conducted online. These research settings are known to have strong replication rates with in-person laboratory studies^1–6^ and are used widely across all cognitive disciplines. Interpretations of task performance from these online studies are often based on the assumption that participants are fully attentive to the cognitive task they are completing. Indeed, response time and accuracy data from various cognitive tasks are commonly taken as indices of the time-course of information processing and the limits of the cognitive system^7–11^. If individuals were found to be engaging in considerable off-task behaviour while participating in online studies, these common assumptions could be called into question.

Studies examining off-task behaviours like media multitasking, defined as multitasking wherein at least one of the tasks is media-based (e.g., working while listening to music, watching TV while texting on a smartphone), have demonstrated that this behaviour is highly ubiquitous and pervasive across numerous activities^12,13^. For example, Voorveld and van der Goot^14^ uncovered that individuals spend about 17 to 31% of their media time engaged in multitasking behaviours, and Ralph and colleagues^15^ recently demonstrated that two-thirds of participants in a laboratory study volitionally chose to media multitask when given the option to do so, with this behaviour occurring despite individuals’ awareness of its negative impact on study performance^15^. As such, media multitasking appears to be a preferred and selected behaviour across different activities; however, thus far, a clear understanding of the degree to which this behaviour occurs within online studies is lacking.

To date, there have only been a handful of smaller studies that have peripherally explored the prevalence of media multitasking in online studies. One such study administered a survey to assess participants’ aggregate behaviours while completing online studies and found the prevalence of media multitasking to be high, ranging from 8 to 20%^16^. Others have indirectly measured this behaviour for data quality or filtering purposes by administering compliance checks that ask participants whether they engaged in any additional activities during their online study^17,18^. This work has found prevalence rates ranging from 14 to 19%. These individual studies suggest that media multitasking within online testing environments does occur to a substantial degree; however, a more comprehensive analysis is needed across larger datasets to uncover the prevalence of this behaviour on a broader scale. Thus, in the present study, we investigated the prevalence of media multitasking within online studies by conducting a secondary data analysis across multiple datasets that queried instances of this behaviour.

## Methods

### Study design and participants

To perform the secondary data analysis, all existing datasets from online studies conducted by the Vision and Attention Laboratory at the University of Waterloo were collated. This laboratory setting was selected due to its inclusion of at least one direct measure of media multitasking behaviour during online studies and due to its use of various experimental and methodological paradigms. All participants were comprised of healthy adults, who were 18 years of age or older, and they were reimbursed for their time with either 1 course credit per hour (if via the University of Waterloo’s Sona virtual participant pool) or approximately USD$ 7.25 per hour (if via Amazon Mechanical Turk marketplace). Participants provided informed consent prior to taking part in the studies. All studies were reviewed and approved for secondary data analysis by the University of Waterloo’s Research Ethics Board, and all studies were conducted in compliance with the Tri-Council Policy Statement. Access to all datasets was obtained in April 2022.

The final sample comprised of 2,972 participants from 16 separate online studies that were collected between May 2016 and April 2022 (see Table 1 for descriptive information). Fourteen studies contained full demographic information, one study contained partial demographic information, and one study had none. Based on the available demographic data, our sample consisted of 1760 women, 988 men, 11 non-binary, 1 two-spirit, and 30 not specified (*M*_age_ = 25.08, *SD*_age_ = 9.80). In total, seven out of 16 studies were classified as video viewing tasks, eight were sustained attention tasks, and one was a collaborative group project task. In addition, eleven out of 16 studies were conducted using Sona virtual participant pool, while five studies were run on Amazon Mechanical Turk marketplace.

**Table 1:** Descriptions of online research studies examining media multitasking behaviours.

#	Dataset	Type of task	Method of recruitment	Date of collection	Sample size	Gender	Age	Primary outcome variable for assessing media multitasking
1	Pereira*, Ayers-Glassey*, Wammes, and Smilek (in prep)	Video Viewing	SONA	Jan 2021–Aug 2021	120	90 women, 26 men, 1 non-binary, 3 not specified	*M* = 20.6, *SD* = 3.8	Q. "While completing this study, were you engaged in any other activities outside of the contents of the experiment (e.g., attending to content in another browser, listening to music, or using a smartphone/tablet while completing the study)?" Options. 1: Yes / 2: No, I didn't engage in any activities outside of the contents of this study
2	Pereira*, Ayers-Glassey*, Wammes, and Smilek (in prep)	Video Viewing	SONA	Apr 2021–Aug 2021	126	75 women, 49 men, 1 non-binary, 1 not specified	*M* = 20.4, *SD* = 2.3	
3	Pereira*, Ayers-Glassey*, Wammes, and Smilek (in prep)	Video Viewing	SONA	May 2021–Oct 2021	116	67 women, 46 men, 1 non-binary, 2 not specified	*M* = 20.8, *SD* = 1.9	
4	Ayers-Glassey, Pereira, Wammes, and Smilek (in prep)	Video Viewing	SONA	Nov 2021–Jan 2022	118	83 women, 30 men, 1 non-binary, 4 not specified	*M* = 19.8, *SD* = 2.2	
5	Pereira, Stuart, Ayers-Glassey, Korst-Fagundes, and Smilek (data collection)	Video Viewing	SONA	Apr 2021–Aug 2021	133	69 women, 58 men, 4 non-binary, 2 not specified	*M* = 20.8, *SD* = 3.1	
6	Park, Smith, Pereira, and Smilek (in prep)	Video Viewing	SONA	Nov 2020–Dec 2020	112	79 women, 33 men	*M* = 19.6, *SD* = 2.1	Q. "Were you doing anything else while also completing this study?" Options. 1: Yes / 2: No
7	Park, Smith, Pereira, and Smilek (in prep)	Video Viewing	SONA	Jun 2021–Oct 2021	184	139 women, 45 men	*M* = 20.3, *SD* = 2.8	
8	Ralph and Smilek (2017)	N-back	M Turk	May 2016–June 2016	317	154 women, 163 men	*M* = 32.9, *SD* = 9.1	Q. "Lastly, given that this study is about media use and multitasking, we are also interested in whether you were multitasking with media while you completed this study. Please answer honestly. You will receive your HIT regardless of your response." Options. 1: I was multitasking during this study / 2: I was not multitasking during this study
9	Ralph, Seli, Wilson, and Smilek (2020)	N-back	M Turk	May 2017	201	86 women, 114 men, 1 not specified	*M* = 33.4, SD = 9.3	Q. "Lastly, given that this study is about media use and multitasking, we are also interested in whether you were multitasking with media while you completed this study. Were you multitasking with any other media, besides the provided video, while you completed the attention task (n-back)? Please answer honestly. You will receive your HIT regardless of your response." Options. 1: Yes / 2: No
10	Stuart, Smith, and Smilek (in prep)	2-back	M Turk	Jan 2020–Mar 2020	180	64 women, 113 men, 1 non-binary, 2 not specified	*M* = 40.0, *SD* = 12.6	Q. "Did you engage in an external secondary task during the study?" Options. 1: Yes / 2: No
11	Stuart, Smith, and Smilek (in prep)	2-back	SONA	May 2021–Jul 2021	191	158 women, 29 men, 2 non-binary, 1 two-spirit, 1 not specified	*M* = 21.3, *SD* = 4.2	
12	Drody, Ralph, Danckert, and Smilek (2022)	2-back	M Turk	Nov 2020–Dec 2020	180	N/A^a^	N/A	Q. "While completing this study, were you engaged in any media-related activities outside of the contents of the experiment (e.g., attending to content in another browser, listening to music or using a smartphone/tablet while completing the study)?" Options. 1: Yes / 2: No, I didn’t engage in any activities outside of the contents of this study / 3: No, but I was engaged in other, media-unrelated activities while completing this study
13	Drody, Ralph, Danckert, and Smilek (in prep)	1-back	SONA	Oct 2020–Nov 2020	302	218 women, 81 men, 3 not specified	*M* = 19.9, *SD* = 3.2	
14	Drody, Ralph, and Smilek (in prep)	1-back	M Turk	June 2021–July 2021	153	64 women, 88 men, 1 not specified	*M* = 39.7 *SD* = 13.2	
15	Marty-Dugas, Ayers-Glassey, and Smilek (in prep)	SART	SONA	Feb 2021 – Apr 2022	452	350 women, 94 men, 8 not specified	*M* = 20.6, *SD* = 5.2	Q. "While completing this study, approximately what percentage of time were you engaged in visual media-related activities outside of the contents of the experiment (e.g., attending to content in another browser window, watching television, or using a smartphone/tablet)?" Options. 0–100 slider scale
16	Bagla, Drody, and Smilek (in prep)	Collaborative Group Project	SONA	Nov 2020	87	64 women, 19 men, 2 not specified^b^	*M* = 20.2, *SD* = 2.5	Q. "Did you access any other apps or web pages, other than those required for this experiment, during the time you participated in this study? (ex. emails, social media platforms such as Facebook, etc.)?" Options. 1: Yes / 2: No

^a^Demographic data was not collected.

^b^Demographic data was not provided by all participants. Therefore, demographic statistics for this study are based on the 85 participants who did respond to the demographic questions.

### Primary outcome variable for assessing media multitasking

To determine the prevalence of media multitasking within online studies, the primary outcome variable consisted of self-report responses to questions that directly probed whether participants had engaged in other activities while completing their task. All questions were administered to participants upon completion of their task and slight variations existed amongst the questions based on wording and response options (see Table 1). To equate all responses within the same construct space, participants were scored as having engaged in media multitasking if they (1) responded ‘Yes’ to media multitasking during the task (for dataset #s 1–14 and 16) or (2) indicated that they spent any length of time (i.e., greater than 0% on the slider scale) engaged in other media-based activities during the study (for dataset # 15).

## Results

### Data analysis

To best assess the overall prevalence of media multitasking within online studies, we conducted a meta-analysis of single proportion data across all datasets^19^. Since there was no a priori reason to assume that the prevalence rate of media multitasking was homogenous across all studies (e.g., due to differences based on participant samples, study design, and primary outcome definitions), we used a random-effects model to account for variability within the datasets^20,21^. Measures of heterogeneity supported this model selection (Cochran’s *Q*(15) = 1027.70, Higgins *I*^2^ = 98.54%).

As per the random-effects model, both the prevalence rate for the population proportion estimates and the standard errors for the study-specific estimates were adjusted by a modified sampling weight (W*), which was based on the reciprocal of the sum of the within-studies variance (*v*) and the between-studies variance (τ^2^). See OSF repository (https://osf.io/bd94j/) for a detailed calculation sheet.

### Prevalence of media multitasking

**Figure 1:**
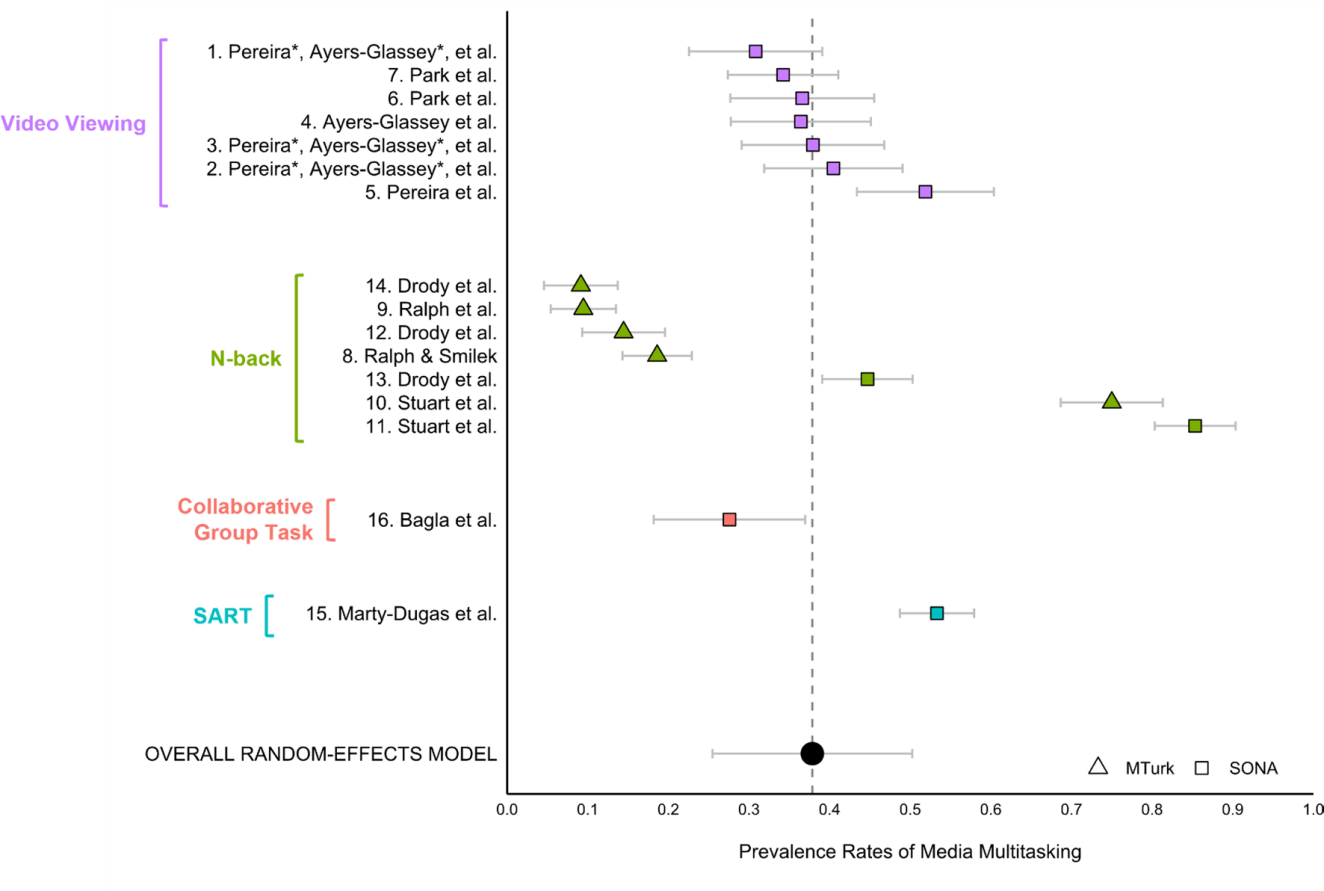
*Prevalence Rates of Media Multitasking across Studies.* Forest plot depicting means and 95% confidence intervals for prevalence rates of media multitasking within each dataset, as well as the point estimate for the overall random-effects model. Coloured labels on the left indicate type of task. Prevalence rates of media multitasking ranged from 0.09 to 0.85, with no differences found across method of recruitment, type of task, length of task, gender of participant, or question phrasing.

Media multitasking occurred frequently, with proportions ranging from 0.09 to 0.85. Notably, there was considerable variability in rates of media multitasking across studies (τ^2^ = 0.06). The random-effects model was significant, with an overall point estimate of 0.38 (95% CI [0.25, 0.50], *p* < 0.001). Figure 1 contains a forest plot illustrating the overall prevalence of media multitasking found within each of the studies and within our random-effects model.

Further analyses revealed that the point estimate did not differ across method of recruitment (SONA = 0.44, 95% CI [0.30, 0.57]; Amazon Mechanical Turk = 0.25, 95% CI [0.06, 0.45], *p* = 0.12), type of task (Video Viewing = 0.38, 95% CI [0.15, 0.61]; N-back = 0.37, 95% CI [0.14, 0.60], *p* = 0.90), length of task (0-30 min = 0.47, 95% CI [0.39, 0.54]; 30-45 min = 0.29, 95% CI [0.20, 0.36]; 45–60 min = 0.38, 95% CI [0.28, 0.48], *p* = 0.35), or gender of participant (women = 0.39, 95% CI [0.24, 0.53]; men = 0.41, 95% CI [0.30, 0.52], *p* = 0.75). In addition, no point estimate differences were found based on whether questions assessed media-related versus general off-task behaviours (i.e., media-related datasets 8, 9, 15, and 16 = 0.27, 95% CI [0.06, 0.48]; general datasets 1–7 and 10–14 = 0.41, 95% CI [0.26, 0.57], *p* = 0.26) nor based on whether questions provided more descriptive information about media multitasking (descriptive datasets 1–5 and 12–16 = 0.35, 95% CI [0.23, 0.46]; non-descriptive datasets 6–11 = 0.14, 95% CI [0.16, 0.70] *p* = 0.46).

In order to check for potential bias across the datasets used for the meta-analysis, we employed a triangulation approach by assessing Begg's rank correlation test^22^, Egger's regression test^23^, and the Luis Furuya-Kanamori (LFK) index^24^. All measures quantify the relationship between a dataset's effect size and a measure of its precision (e.g., study size, standard error, absolute z-score) across the meta-analysis, with a lack of bias illustrated as a lack of association or lack of asymmetry between the two indices. Within the current analysis, no evidence of association or asymmetry was found across any of the bias measures (Begg's *p* = 0.63; Egger's *p* = 0.36; LFK index = 0.76). Accompanying plots depicting bias measures can be found in “Supplementary Materials”.

## Discussion

We explored the prevalence of media multitasking during online studies by conducting a secondary data analysis of 16 studies, encompassing nearly 3,000 participants in total. We found that media multitasking occurred frequently in these studies, with rates averaging 38%, and ranging from a low of 9% in some cases to a high of 85% in others. These findings are consistent with past studies that have examined the presence of media multitasking during online studies^16^, and further extend this literature by demonstrating that when participants are left to self-direct their own attention, media multitasking occurs to a much higher degree than previously expected.

These findings demonstrate a concerning violation of the fundamental assumption held by many when conducting experimental tasks: namely, that participants are devoting or attempting to devote their attention fully to the experimental task before them. Because online testing environments forgo the strict experimental control that researchers have in the laboratory, researchers have had to rely on an agreement between themselves and participants—sometimes explicit and other times tacit—that participants will devote their full attention to the experimental task-at-hand and eliminate or limit any off-task behaviours. However, our findings illustrate that this agreement is not fulfilled by many participants. Instead, some participants seem to approach the task with a different mindset, perhaps understanding that their obligations are fulfilled if they only devote *some* attention to the experimental task.

Given the striking level of media multitasking found during online studies, our results raise questions about the conclusions that can be drawn from online cognitive studies, as well as the reliability and generalizability of their findings. That is, it is possible that online studies reflect the cognitive processes of individuals when they are in a disengaged state and that the results obtained from such studies do not extend to contexts in which individuals are more fully attentive. Indeed, testing participants while they are disengaged from the experimental task is unlikely to yield stable or accurate estimates of the time course of mental processing or the capacity limits of cognition. Furthermore, given that some cognitive tasks are used as measures of individual trait-like cognitive abilities —e.g., the *n*-back task is used to index working memory ability^25^— it is possible that individual differences in task performance during online studies might be confounded by individual differences in participants’ tendencies to media multitask. Although disengagement may be less of a concern when studying cognition that is either unhindered by off-task behaviours (e.g., during decision-making studies) or emboldened by it (e.g., during real world study designs), our increased reliance on online forms of data collection over the years highlight the importance and necessity of measuring off-task behaviours when studying cognition during online studies.

Our specific concerns about participant inattentiveness during online studies may at first blush seem inconsistent with the overlap in outcomes for studies performed across online and in-laboratory testing environments^1–6^; after all, if online and in-laboratory studies yield the same behavioural and performance outcomes, it would seem that inattention during online studies can safely be ignored. However, it is important to note that prior work has demonstrated that participant disengagement also occurs in laboratory settings. For instance, there is a growing body of work showing that during in-person laboratory studies, participants disengage in various ways, including by mind-wandering^26^, mind-blanking^27^, and attending to external distractions present in the testing environment^28^. In-person laboratory studies have revealed that people mind wander approximately 30%^29^ to 70%^30^ of the time, which is roughly similar to the media multitasking rates reported here. Thus, it would appear that we should be equally concerned about inattention across both online and in-laboratory contexts. It may be the case that media multitasking during online studies may be replacing mind-wandering to some extent. This notion is supported by Ralph and colleagues^31^ who found that when participants were given the option to media multitask during a cognitive task, they reported a decrease in mind-wandering behaviours that was tied to an increase in media multitasking. It appears that when individuals are allowed to simultaneously engage in other activities, a subset of those who would normally mind-wander during cognitive tasks instead prefer to media multitask. Although more work is needed to understand the degree of overlap between media multitasking in online settings and mind-wandering in the laboratory, it seems that disengagement from experimental tasks is a broadly applicable concern across both forms of testing environments.

A point worth noting is that disengagement during online studies may be particularly problematic because these environments allow participants to engage in a greater variety of off-task activities (e.g., using one’s cellphone or watching TV^16,32^) than would normally be permitted in the laboratory. These different multitasking combinations can have differential effects on one’s ability to perform a task given that sharing cognitive resources between an auditory and visual task (e.g., listening to music while completing an attentional task) is likely to impact performance to a lesser degree than sharing resources between two visual tasks (e.g., watching a video while completing an attentional task^33^). This greater variety of activities may have more varied effects on participant performance, suggesting that disengagement during online studies may introduce more uncontrolled noise into one’s data than disengagement in the laboratory.

When considering our results, it is important to acknowledge the meta-analytic decisional criteria that was applied in the current study that may have impacted the prevalence rates found for media multitasking. On a construct level, we used self-reported instances of media multitasking during various tasks as our primary outcome variable; however, it is possible that differences in the phrasing or interpretation of the media multitasking question may have led to variance in this measure. For example, it is unclear whether participants who engaged in very short or infrequent bouts of media multitasking (e.g., checking their phone for messages) responded similarly to participants who engaged in media multitasking throughout the task (e.g., listening to music), particularly given that a majority of questions only provided for binary yes/no response options rather than continuous slider scaling. Although our subgroup analyses demonstrated that prevalence rates of media multitasking did not significantly differ based on the phrasing of the media multitasking questions (i.e., whether or not media multitasking was explicitly mentioned and whether or not examples of media multitasking were provided), future work would benefit from assessing media multitasking based on the *amount* that participants media multitasked to provide clarity for participants and greater exploratory power for researchers.

In addition, on a selection level, all of the data used in our meta-analysis was taken from studies conducted within a single research group. Although this might raise concerns surrounding the generalizability of the results beyond the studies examined in the present work, there are several reasons to believe that our findings would extrapolate to other online research settings/studies. Most importantly is the large sample that is reflected within the analyses, which captures diversity across testing periods (pre- and during pandemic), experimental tasks (passive viewing and sustained attentional), participant samples (SONA and Amazon Mechanical Turk), and participant demographics (63% women, 35% men; age range of 16–73 years). This diversity is likely to enhance the degree to which our findings would align with rates of media multitasking during other online studies in the field. Furthermore, measures of heterogeneity (i.e., Cochran’s *Q*, Higgin’s *I*^2^) strongly supported the notion that there was a high degree of variability across the datasets used in the meta-analysis, which converged with measures of bias (i.e., Begg’s rank correlation, Egger’s regression, LKF index). Together, these indirect and direct metrics provide evidence of the breadth of media multitasking during online studies that was captured within the meta-analysis. More research examining nuances across studies, tasks, and demographic groups would certainly be a welcome addition to the literature.

Other interesting avenues for future research on media multitasking could concern the temporal dynamics of this behaviour, and whether participants engaged in media multitasking consistently throughout the entire experiment or flexibly during certain parts of the task only. Although past work provides evidence to suggest that media multitasking increases with time on task^34^, the current study only assessed media multitasking as a holistic subjective measure rather than a moment-to-moment objective measure. For this reason, using temporally specific paradigms could provide insight into how individuals engage in this behaviour on a moment-to-moment basis. Some researchers have taken this approach by providing participants with the option to media multitask on a trial-by-trial basis during their study^15,17,18^; other viable options could involve tracking computer-based off-task behaviours that may be indicative of media multitasking, such as changes in screen size or navigation away from the study page (see^35^). These approaches could additionally shed light on how performance varies with media multitasking throughout the task and the degree to which these two measures can be linked over time.

Another intriguing question is why participants would engage in media multitasking given that previous research has demonstrated that they are often aware of the negative performance costs of engaging in this behaviour^15^. One potential explanation might relate to the value participants place on completing online studies. According to this account^36^, there is a trade-off between engaging in a current activity and the possible completion of other tasks, and factors such as boredom and mental effort may signal to participants the opportunity costs of engaging in their current activity. Once these factors reach an individual threshold, participants may be motivated to engage in other activities instead, even to the detriment of the current task’s performance. Consistent with this perspective, Wiradhany and colleagues^37^ have proposed an attentional framework wherein attention passes through phases of exploitation and exploration, the former of which allows individuals to fully engage with a task and the latter of which allows individuals to search for alternative activities. As such, individuals may engage in media multitasking as a form of exploration when the benefits of exploiting their current task begin to fade. Consistent with this notion, it has been shown that increasing participant motivation (i.e., manipulating the perceived opportunity costs of or the rewards associated with engaging in an activity) reduces media multitasking in laboratory settings^38^. Therefore, the degree to which each individual values participating in a particular online study may influence the perceived costs and benefits of completing the study versus engaging in other activities, which may ultimately influence whether a participant chooses to media multitask.

The current pandemic has resulted in the increased use of online studies, which has allowed researchers to collect data efficiently using large and diverse samples. Often this comes at the cost of reduced experimental control, leaving open the possibility that participants may simultaneously engage in off-task activities. Our current results demonstrate that off-task activities like media multitasking occur frequently during online studies, raising significant questions about the interpretation of performance in terms of cognitive processes within online testing environments.

## Supplementary Information

Supplementary Information.

## Data Availability

The data as well as the materials used for the calculations of results for this study are publicly accessible at https://osf.io/bd94j/. There was no pre-registration for this study.

## References

[1] Casler K, Bickel L, Hackett E (2013). Separate but equal? A comparison of participants and data gathered via Amazon’s MTurk, social media, and face-to-face behavioral testing. Comput. Hum. Behav..

[2] Claypoole VL, Neigel AR, Fraulini NW, Hancock GM, Szalma JL (2018). Can vigilance tasks be administered online? A replication and discussion. J. Exp. Psychol. Hum. Percept. Perform..

[3] Crump MJ, McDonnell JV, Gureckis TM (2013). Evaluating Amazon's Mechanical Turk as a tool for experimental behavioral research. PloS One.

[4] Giraudier M, Ventura-Bort C, Wendt J, Lischke A, Weymar M (2022). Memory advantage for untrustworthy faces: Replication across lab-and web-based studies. Plos One.

[5] Paolacci G, Chandler J, Ipeirotis PG (2010). Running experiments on Amazon Mechanical Turk. Judgm. Decis. Mak..

[6] Stothart CR, Boot WR, Simons DJ (2015). Using Mechanical Turk to assess the effects of age and spatial proximity on inattentional blindness. Psychol: Collabra.

[7] Jensen AR (2006). Clocking the Mind: Mental Chronometry and Individual Differences.

[8] Medina JM, Wong W, Díaz JA, Colonius H (2015). Advances in modern mental chronometry. Front. Hum. Neurosci..

[9] Pashler H (1994). Dual-task interference in simple tasks: Data and theory. Psychol. Bull..

[10] Posner MI (1978). Chronometric Explorations of Mind.

[11] Sternberg S (1969). The discovery of processing stages: Extensions of Donders' method. Acta Psychol..

[12] Junco R, Cotten SR (2012). No A 4 U: The relationship between multitasking and academic performance. Comput. Educ..

[13] Moreno MA (2012). Internet use and multitasking among older adolescents: An experience sampling approach. Comput. Human Behav..

[14] Voorveld HAM, van der Goot M (2013). Age differences in media multitasking: A diary study. J. Broadcast. Electron. Media.

[15] Ralph BCW, Seli P, Wilson KE, Smilek D (2020). Volitional media multitasking: Awareness of performance costs and modulation of media multitasking as a function of task demand. Psychol. Res..

[16] Necka EA, Cacioppo S, Norman GJ, Cacioppo JT (2016). Measuring the prevalence of problematic respondent behaviors among MTurk, campus, and community participants. PLoS One.

[17] Drody AC, Ralph BCW, Danckert J, Smilek D (2022). Boredom and media multitasking. Front. Psychol..

[18] Ralph BCW, Smilek D (2017). Individual differences in media multitasking and performance on the n-back. Atten, Percept, Psychophys.

[19] Khan S (2020). Meta-Analysis: Methods for Health and Experimental Studies.

[20] Borenstein M, Hedges LV, Higgins JP, Rothstein HR (2010). A basic introduction to fixed-effect and random-effects models for meta-analysis. Res. Synth. Methods.

[21] Higgins JPT, Thompson SG, Spiegelhalter DJ (2009). A re-evaluation of random-effects meta-analysis. J. R. Stat. Soc. Ser. A Stat. Soc..

[22] Begg CB, Mazumdar M (1994). Operating characteristics of a rank correlation test for publication bias. Biometrics.

[23] Egger M, Smith GD, Schneider M, Minder C (1997). Bias in meta-analysis detected by a simple, graphical test. Br. Med. J..

[24] Furuya-Kanamori L, Barendregt JJ, Doi SAR (2018). A new improved graphical and quantitative method for detecting bias in meta-analysis. Int. J. Evid. Based Healthc..

[25] Kirchner WK (1958). Age differences in short-term retention of rapidly changing information. J. Exp. Psychol..

[26] Smallwood J, Schooler JW (2006). The restless mind. Psychol. Bull..

[27] Ward AF, Wegner DM (2013). Mind-blanking: When the mind goes away. Front. Psychol..

[28] Stawarczyk D, Majerus S, Maj M, Van der Linden M, D'Argembeau A (2011). Mind-wandering: Phenomenology and function as assessed with a novel experience sampling method. Acta Psychol..

[29] Jin CY, Borst JP, van Vugt MK (2019). Predicting task-general mind-wandering with EEG. Cogn. Affect. Behav. Neurosci..

[30] Seli P, Schacter DL, Risko EF, Smilek D (2017). Increasing participant motivation reduces rates of intentional and unintentional mind wandering. Psychol. Res..

[31] Ralph BCW, Smith AC, Seli P, Smilek D (2019). Yearning for distraction: Evidence for a trade-off between media multitasking and mind wandering. Can. J. Exp. Psychol..

[32] Clifford S, Jerit J (2014). Is there a cost to convenience? An experimental comparison of data quality in laboratory and online studies. J. Exp. Polit. Sci..

[33] Hwang Y, Jeong SH (2018). Multitasking and task performance: Roles of task hierarchy, sensory interference, and behavioral response. Comput. Hum. Behav..

[34] Wammes JD, Ralph BCW, Mills C, Bosch N, Duncan TL, Smilek D (2019). Disengagement during lectures: Media multitasking and mind wandering in university classrooms. Comput. Educ..

[35] Permut S, Fisher M, Oppenheimer DM (2019). TaskMaster: A tool for determining when subjects are on task. Adv. Meth. Pract. Psychol. Sci..

[36] Kurzban R, Duckworth A, Kable JW, Myers J (2013). An opportunity cost model of subjective effort and task performance. Behav. Brain Sci..

[37] Wiradhany W, Baumgartner S, de Bruin A (2021). Exploitation–exploration model of media multitasking. J. Media Psychol..

[38] Ralph BCW, Smith AC, Seli P, Smilek D (2021). The relation between task-unrelated media multitasking and task-related motivation. Psychol. Res..

