# Supplementary material for: A desire for distraction: uncovering the rates of media multitasking during online research studies
Source: Sci Rep. 2023 Jan 16;13:781. doi: 10.1038/s41598-023-27606-3 (PMC9842732; doi:10.1038/s41598-023-27606-3)
Supplement: Supplementary file 1 — Supplementary Information. [file 41598_2023_27606_MOESM1_ESM.docx]

Supplementary Materials for

**A desire for distraction: Uncovering the rates of media multitasking during online research studies**

Allison C. Drody^*^, Effie J. Pereira, & Daniel Smilek

Department of Psychology, University of Waterloo, Waterloo, Ontario, Canada

^*^Correspondence to:

Allison Drody

Email: acdrody@uwaterloo.ca

**Results**

Figure S1a depicts a funnel plot [1], which illustrates a scatterplot of the standard error for each of the studies included in the meta-analysis against its observed prevalence. Funnel plots that depict symmetry around the vertical line provide evidence for no overall bias in the meta-analysis. However, since funnel plots can be misleading when assessing prevalence data [2-4], Figure S1b depicts the Doi plot [5-6], which illustrates a continuous curve depicting the absolute z-scores of the observed prevalence for each of the studies included in the meta-analysis against the observed prevalence. Doi plots that depict general symmetry provide evidence for no overall bias in the meta-analysis.

**Figure S1.**

*Funnel plot (a) and Doi plot (b) depicting a lack of bias within the meta-analysis*


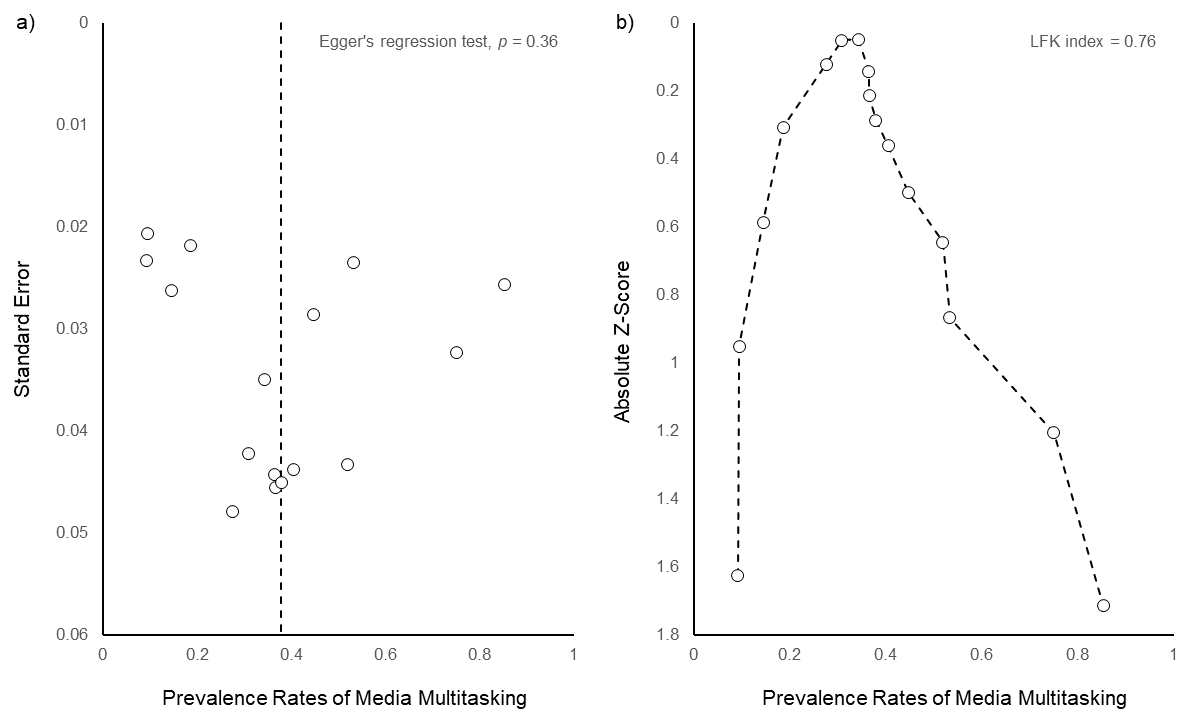


**References**

1. Egger, M., Smith, G. D., Schneider, M., & Minder, C. Bias in meta-analysis detected by a simple, graphical test. *Br Med J*, **315**, 629–634 (1997).

2. Cheema, H. A., Shahid, A., Ehsan, M., & Ayyan, M. The misuse of funnel plots in meta-analyses of proportions: Are they really useful?. *Clin Kidney J*, **15**, 1209-1210 (2022).

3. Hunter, J. P., Saratzis, A., Sutton, A. J., Boucher, R. H., Sayers, R. D., & Bown, M. J. In meta-analyses of proportion studies, funnel plots were found to be an inaccurate method of assessing publication bias. *J Clin Epidemiol*, **67**, 897-903 (2014).

4. Tang, J. L., & Liu, J. L. Misleading funnel plot for detection of bias in meta-analysis. *J Clin Epidemiol*, **53**, 477-484 (2000).

5. Doi, S. A. Rendering the Doi plot properly in meta-analysis. *Int J Evid Based Healthc*, **16**, 242-243 (2018).

6. Furuya-Kanamori, L., Barendregt, J. J., & Doi, S. A. R. A new improved graphical and quantitative method for detecting bias in meta-analysis. *Int J Evid Based Healthc*, **16,** 195–203 (2018).
